# Review of Ligand Specificity Factors for CYP1A Subfamily Enzymes from Molecular Modeling Studies Reported to-Date

**DOI:** 10.3390/molecules22071143

**Published:** 2017-07-08

**Authors:** Jayalakshmi Sridhar, Navneet Goyal, Jiawang Liu, Maryam Foroozesh

**Affiliations:** Department of Chemistry, Xavier University of Louisiana, 1 Drexel Dr., New Orleans, LA 70125, USA; jsridhar@xula.edu (J.S.); ngoyal@xula.edu (N.G.); jliu1@xula.edu (J.L.)

**Keywords:** cytochrome, P450 1A1, P450 1A2, docking, quantitative structure activity studies (QSAR), molecular modeling, dynamics, active site

## Abstract

The cytochrome P450 (CYP) family 1A enzymes, CYP1A1 and CYP1A2, are two of the most important enzymes implicated in the metabolism of endogenous and exogenous compounds through oxidation. These enzymes are also known to metabolize environmental procarcinogens into carcinogenic species, leading to the advent of several types of cancer. The development of selective inhibitors for these P450 enzymes, mitigating procarcinogenic oxidative effects, has been the focus of many studies in recent years. CYP1A1 is mainly found in extrahepatic tissues while CYP1A2 is the major CYP enzyme in human liver. Many molecules have been found to be metabolized by both of these enzymes, with varying rates and/or positions of oxidation. A complete understanding of the factors that govern the specificity and potency for the two CYP 1A enzymes is critical to the development of effective inhibitors. Computational molecular modeling tools have been used by several research groups to decipher the specificity and potency factors of the CYP1A1 and CYP1A2 substrates. In this review, we perform a thorough analysis of the computational studies that are ligand-based and protein-ligand complex-based to catalog the various factors that govern the specificity/potency toward these two enzymes.

## 1. Introduction

Research on cytochrome P450 enzymes has evolved over the years from the initial in-vitro substrate metabolism studies to the studies on the metabolism of drugs and the role of these enzymes in various diseases including cancer. The Human Genome Project revealed that there are 57 human P450 (CYP) genes and 58 pseudogenes [1]. CYP enzymes are found in all living organisms and catalyze the mono-oxygenation of many different substrates. Mammalian cytochrome P450 enzymes can oxidize both endogenous compounds and xenobiotics, thereby playing critical roles in cholesterol and hormone synthesis, metabolism of endogenous compounds such as vitamin D, drug deactivation, and xenobiotics detoxification. The CYP1 family of P450 enzymes comprises of three members 1A1, 1A2, and 1B1. CYP1A1 and CYP1A2 are closely related with the CYP1A gene cluster that is mapped to chromosome 15q24.1, with head-to-head orientation, sharing a 23 kb bi-directional promoter and a common 5′-flanking region [2]. CYP1A1 gene spans 5.8 kb and CYP1A2 gene spans 7.8 kb with seven exons and six introns [3,4]. CYP1A2 is the highly expressed cytochrome enzyme in the human liver (~13–15%) [5], while CYP1A1 expression levels are low in the liver (<0.7%), and it is mostly found in extrahepatic tissues [6,7].

## 2. CYP1A Substrates

The span of enzymatic activities of CYP1A1 and CYP1A2 vastly overlaps with hydroxylations and oxidations of aromatic compounds including polycyclic aromatic hydrocarbons. Arachidonic acid and eicosapentoic acid, involved in synthesis of inflammation factors and hormones such as melatonin and 17β-estradiol, are endogenous substrates for CYP1A1 [8,9,10]. CYP1A1-dependent metabolic activation of procarcinogens into carcinogens via epoxides is well established in cancer initiation [11,12]. Other CYP1A1 exogenous substrates include heterocyclic aromatic amines of industrial origin or those that are found in burnt food such as meat, fuel combustion products, and tobacco products [12,13]. Some of the endogenous substrates that are metabolized by both CYP1A1 and CYP1A2 include melatonin, 6-hydroxylate melatonin, and arachidonic acid, etc. Several other endogenous substrates are metabolized by CYP1A2 including estradiol, estrone, bilirubin, and uroporphyrinogen, etc. CYP1A2 metabolizes numerous natural products that result in toxic products such as transformation of methyleugenol to 1′-hydroxymethyleugenol, estragole to reactive metabolites, oxidation of nephrotoxins, and aristolochic acids [14,15,16]. CYP1A2 plays an important role in the metabolism of several clinical drugs including analgesics, antipyretics, antipsychotics, antidepressants, anti-inflammatory, and cardiovascular drugs [17,18,19,20].

## 3. Structure and Catalytic Cycle

**Figure 1:**
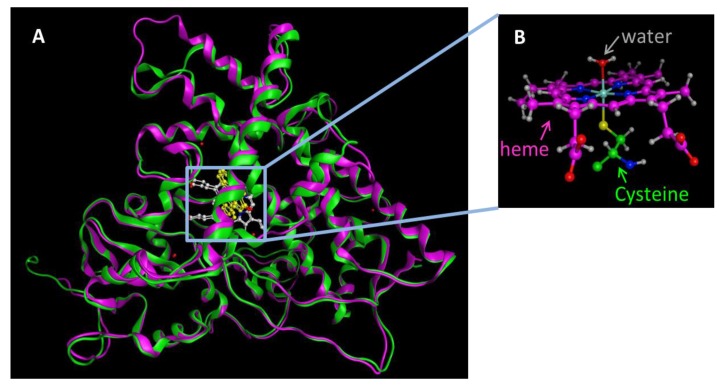
(**A**) The X-ray crystal structures of CYP1A1 (4I8V.pdb, pink) and CYP1A2 (2HI4.pdb, green) are superposed as ribbon model. Atoms are shown as ball and stick models. Heme is colored white and the substrate flavone is colored yellow. The total rmsd for these structures is 1.4 Å; (**B**) Native state of the heme bound to Cys sulfur atom as the fifth ligand and the water molecule as the sixth ligand.

The overall fold structure of all of the cytochrome P450 enzymes is identical, even though the sequence identify across the entire superfamily is less than 20%. The three-dimensional structure consists of 12 α-helices, A-L, that form the bulk of the protein, and four β-sheets. The heme cofactor is housed within the L-helix and the highly conserved I-helix that is perpendicular to the F/G segment comprising of the F-helix, F/G-loop and the G-helix. The heme Fe atom is bound to the sulfur atom of the Cys in the adjacent loop that has the highly conserved sequence FxxGx(HRK)xCxG. Substrate access and specificity is governed by B-C and F-G helices. The sequence identity between the two CYP1A1 (512 aa) and CYP1A2 (515 aa) enzymes is 71%. The X-ray crystal structure of CYP1A2 (2HI4) was reported by Johnson’s group in 2006 [21] and the CYP1A1 X-ray structure (PDB code: 4I8V) was reported by Scott’s group in 2013 [22]. The two CYP1A enzymes exhibit an overall similarity in their three-dimensional structures (Figure 1). However, there are some key structural differences as exemplified by their X-ray crystal structures (PDB codes 4I8V and 2HI4 for CYP1A1 and CYP1A2, respectively). Scott et al. reported the structural variations between the two enzymes. CYP1A1 and CYP1A2 differ from other cytochrome P450 enzymes in the rigidity of the H/I loop, the break in the F helix, and the secondary structure of the β_4_ region. The H/I loop of CYP1A2 contains two short β-strands made of two residues each, that are not seen in the crystal structure of CYP1A1. The most important structural difference is seen in the F helices of CYP1A1 and CYP1A2. There is a three-residue break in the middle of the F helix in the CYP1A2 structure and this break is extended to five residues in the CYP1A1 structure. The F helix plays an important role in substrate access/egress and is also involved in the binding site regioselectivity and stereoselectivity preferences. The secondary structure adopted by the β_4_ system of two strands is absent in the structures of CYP1A1 and CYP1A2. The turn of the β_4_ loop that lines the binding cavity is known to exhibit ligand interactions and provide ligand access. In addition to these differences, the B’ helix exhibits minor differences in hydrogen bonding pattern between the CYP1A1 and CYP1A2. The notable differences in the features that define the substrate access/regress paths and the substrate-binding cavity indicate that the CYP1A enzymes may indeed allow for substrate specificity in binding, orientation and the point of oxidation.

**Figure 2:**
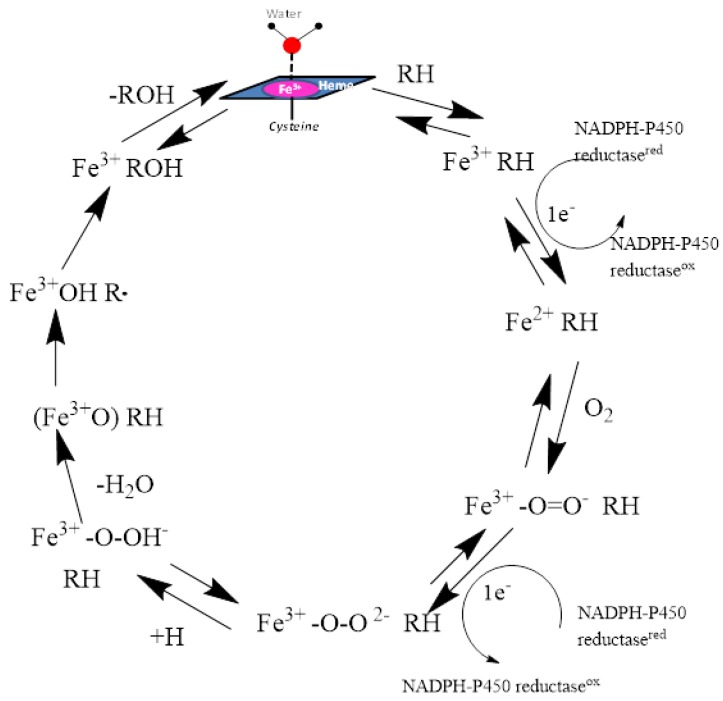
The catalytic cycle of oxidation of substrates by CYP enzymes is shown. The heme iron atom is anchored to the enzyme through the entire cycle by its covalent linkage to the sulfur atom of the Cys residue.

The catalytic cycle of the cytochrome P450 enzymes can be commonly defined by the protein heme iron—oxygen roles through the cycle. The resting state of the enzyme involves a low-spin ferric enzyme with a water molecule coordinated as the sixth ligand of the heme iron atom. The binding of a substrate to the enzyme sometimes changes it to a high-spin ferric state for the enzyme-substrate complex. The next step in the cycle is the oxygen binding to the heme iron to form an oxy-iron-P450 complex which is a stable intermediate. The next sequence of steps occurs rapidly to form unstable intermediates: reduction of the oxy-iron-P450 complex to form the peroxo-ferric-P450 complex, protonation of the distal oxygen to form the hydroperoxo-iron-P450 complex, and a second protonation of the same oxygen followed by heterolytic cleavage of the bond between the two oxygen atoms leading to the formation of a reactive high-valent iron-oxo-P450 complex which is generally called ‘Compound **1**’. Compound **1** then abstracts a hydrogen from the substrate with subsequent radical recombination to produce the oxidized substrate that is egressed. The resting state of the P450 enzymes is regenerated by the binding of a water molecule to the heme iron atom (Figure 2).

## 4. Inhibition of CYP1A Enzymes

The role of CYP1A1 and CYP1A2 in metabolizing procarcinogens to carcinogens leading to mutagenesis and tumorigenesis has been well documented [13,23,24,25,26,27]. Targeting these enzymes for inhibition can help in cancer prevention by inhibiting the conversion of environmental procarcinogens to their carcinogenic forms, inhibiting the formation of carcinogenic hormone derivatives from hormonal precursors, and inhibiting the metabolic inactivation of several therapeutic agents including anticancer drugs [28]. Several small natural compounds are also inhibitors of CYP1A1 and CYP1A2. The quinazolinecarboline alkaloids- evodiamine, dehydroevodiamine and rutaecarpine from *Evodia rutaecarpa,* have been traditionally used for the treatment of hypertension, and gastrointestinal disorders in Chinese medicine [29]. Naturally occurring flavonoids are well known for their inhibition of toxicological processes and drug disposition. These natural inhibitors of CYP1A1 and CYP1A2 could have an important role in cancer prevention by reducing the metabolism of procarcinogens by these enzymes. Thus, they have been prescribed as essential dietary components by regulating agencies worldwide.

The inhibitors of P450 enzymes fall into two main categories- direct competitive inhibitors and time-dependent inhibitors. Competitive inhibitors are capable of accessing the active site and reversibly binding to the active site. These kinds of molecules need to have a much higher affinity to the target enzyme than the natural substrates. Time-dependent inhibitors are also capable of accessing the active site and binding to the active site [30,31]. When these inhibitors are initially incubated with the enzyme before the addition of the substrate, an increase in inhibition is observed, which is a kinetic phenomenon. This category can be further defined by its subset of mechanism-based inactivation wherein the bound inhibitor is oxidized by the enzyme to a highly reactive intermediate that subsequently binds to a reactive amino acid in its proximity. This process permanently changes the enzyme active site, resulting in the inactivation of the enzyme. This process is both time- and cofactor-dependent. Several classes of inhibitors have been found that act as direct competitive inhibitors or time-dependent inhibitors.

## 5. Substrate Binding Site Characteristics

**Figure 3:**
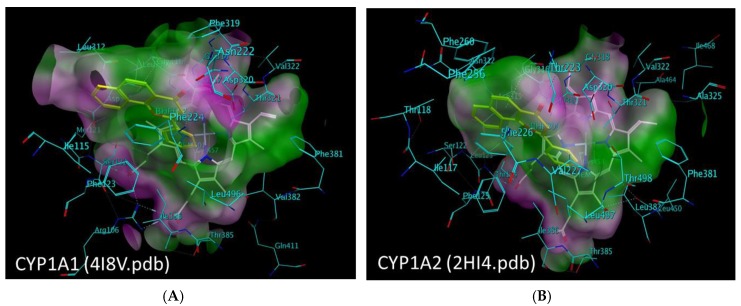
The molecular surface representation of the active site pocket of the (**A**) CYP1A1 and (**B**) CYP1A2 enzymes colored by lipophilicity where the pink region depicts hydrophilic region of the pocket and the green region depicts the lipophilic region of the pocket. The heme residue is represented as white stick model, the ligand (α-naphthoflavone) is shown as yellow stick model, and the enzyme residues are shown as cyan stick models.

The substrate binding cavity is defined by the I, F, G, C and B’ helices, the loop between the K helix and β_1–4_ sheets and the residues at the turn of the β_4_ region. The X-ray crystal structures of the CYP1A1 and CYP1A2 demonstrate several similarities between the two enzymes’ active sites (Figure 3).

A comparative protein structural analysis between the X-ray crystal structures of CYP1A1 and CYP1A2 has been performed by Kesharwani et al. [32,33]. They describe several differences in the six identified substrate recognition sites between the two CYP1A enzymes. They have also identified the residues in CYP1A1 and CYP1A2 showing higher B-factor values than the average B-factor. They are- Asn221, Leu254, Asp320 and Lys499 in the F, G, I and L helices of CYP1A1, and Thr118, Asp320, Thr321, Leu382 and Ile386 in the B’ and I helices and the loop connecting K helix to β_2_ sheet of CYP1A2. Several identical residues are aligned in identical orientations in the active site spaces such as the Ile-115/117, Phe-123/125, Phe-224/226, Thr-321, Asp-320, Ile-386, Leu-496/497, Asn-255/257, and Thr-497/498 in CYP1A1/CYP1A2. The two non-conserved residues with similar properties in the active sites of CYP1A1/CYP1A2 are the Ser116/Thr118 and the Ser122/Thr124. The three non-conserved residues with different properties in the active sites of CYP1A1/CYP1A2 are the Asn222/Thr223, the Leu312/Asn312, and the Val382/Leu382. The B-factor analysis indicated that the non-conserved residues and the residues with higher B-factors demonstrated greater mobility and flexibility.

## 6. Ligand-Based Studies on Isoform Selectivity

While the X-ray crystal structures provide a detailed map of the substrate recognition sites and the active sites, the wide range of the substrates and inhibitors for the two enzymes with varied shapes and sizes indicate the plasticity of the active sites defined by the flexibility and movement of the helices surrounding the active sites. The shapes of the active sites are defined by the F and I helices in the two enzymes CYP1A1 and CYP1A2, forming a flat surface between these helices, that clearly indicate the preference for planar molecules. The selective inhibition of either CYP1A1 or CYP1A2 has been a challenge due to the large number of substrates with varied structures that are metabolized by both enzymes.

**Figure 4:**
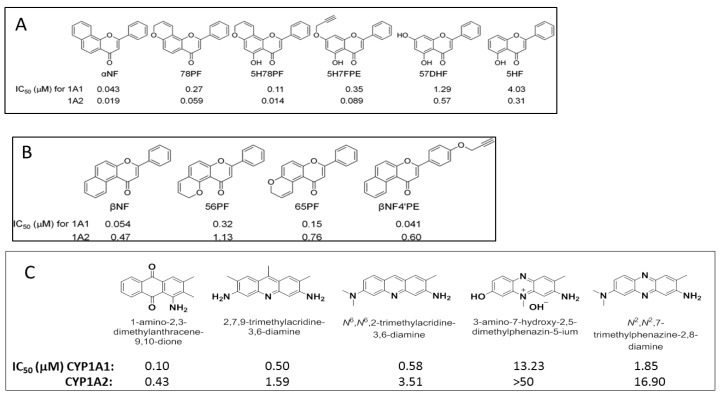

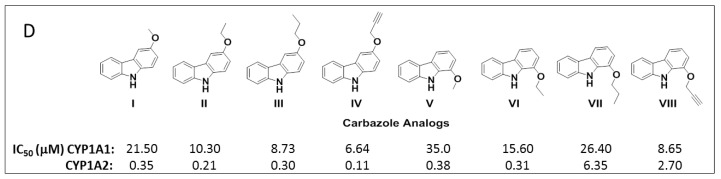
Structures and CYP1A1 & CYP1A2 inhibition data for (**A**) & (**C**) linear planar molecules and (**B**) & (**D**) triangular planar molecules [(**A**) and (**B**) reprinted from reference 34 with permission ].

**Figure 5:**
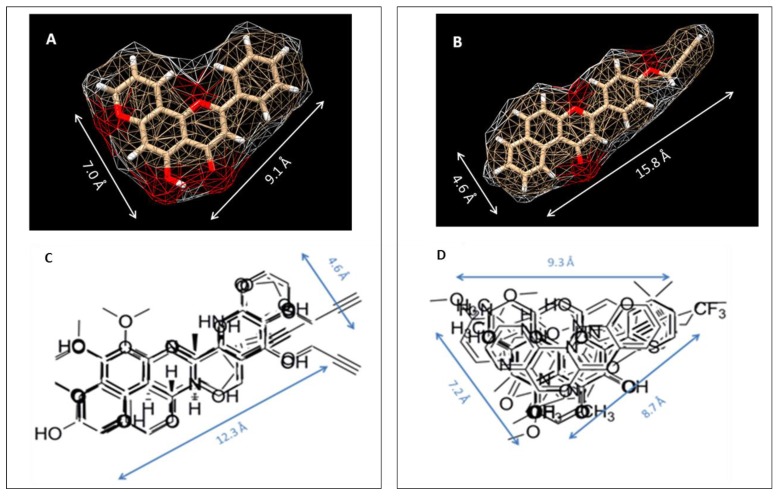
Alignment images of group 1 and group 2 inhibitors. (**A**) and (C) Group 1 represents selective P450 1A1 inhibitors. (**B**) and (D) Group 2 represents selective P450 1A2 inhibitors. [Figure was reprinted from reference 34 with permission].

To study the nature of the active site cavities in these enzymes, a series of planar molecules, naphthoflavones and pyranoflavones, were synthesized by our group. A meta-analysis of these molecules was performed to study the inhibitor selectivity toward CYP1A1 and CYP1A2 (Figure 4). In light of the variations in the inhibitory data from different methods of determination, a selectivity ratio which is a measure of the ratio of the IC_50_ values of the inhibitors for CYP1A1 and CYP1A2 was used to define two groups of inhibitors (IC_50_ for CYP1A1/IC_50_ for CYP1A2 ratios <0.2—group1 and >5—group 2) for the comparative meta-analysis (Figure 5).

Structural alignments between the two groups of inhibitors showed that selective CYP1A1 inhibitors have a long strip shape with an average length of 12.3 Å, and average width of 4.6 Å, showing a narrow and long cavity for CYP1A1. The alignment of the groups of selective CYP1A2 inhibitors form a triangular shape with side lengths of 9.3 Å, 8.7 Å, and 7.2 Å, that are indicative of a narrow compact triangular cavity for CYP1A2 [34]. The impact of the ligand shape on the enzyme specificity for CYP1A1 and CYP1A2 was confirmed by the study of the derivatives of long planar molecules such as anthracene, phenazines, phenothiazines, acridines, and anthracene-9,10-diones, all showing greater inhibition potency for CYP1A1 than CYP1A2 [34,35,36,37] (Figure 4). On the other hand, triangular shaped molecules such as phenanthrenes and carbazoles clearly show greatly increased potency toward CYP1A2 compared to CYP1A1 [34,36,37] (Figure 4).

Several structure-activity relationship studies such as CoMFA, SAR, 3D-QSAR have been undertaken by researchers to understand the ligand interactions with the residues in the protein active site. The goals of such studies included virtual screening, prediction of the site of metabolism, and prediction of the potency/selectivity of inhibition of the CYP enzymes. Our group has recently published extensive reviews on these studies [35,38]. Two types of QSAR models have been employed to study the P450 substrates. The first one is a global QSAR model that is used to predict the ADMET (absorption, distribution, metabolism, elimination and toxicity), which is not very useful in terms of understanding the active site properties and prediction of potency/selectivity. The second type is the detailed QSAR for specific P450 enzymes of smaller sets of molecules that are structurally related. These models are very robust and give a more precise representation of the active site interactions and structural features that impart potency and specificity. Several descriptors including electrostatic, hydrophobic, hydrogen bond acceptor/donor, steric, and desolvation free energy have been studied using quantum/molecular mechanical calculations. Many of these models have been verified by docking studies.

QSAR studies on CYP1A1 substrates have been performed by several research groups on polyaromatic hydrocarbons, flavonoids, benzoxazoles, benzothiazoles, and several other inhibitors of CYP1A1 [39,40,41,42,43,44,45,46]. These studies have shown that the properties that impact the binding of the substrates are the linear planarity of the molecules, the HOMO energy, π-π stacking with the Phe residues, optimally placed acceptor and donor substituents that can interact with the polar residues in the active site, and desolvation (ClogP).

QSAR analysis of CYP1A2 substrates and inhibitors has been widely studied by many researchers as well [39,45,46,47,48,49,50,51]. Chemometric tools that employ stepwise multiple linear regression, genetic function approximation, and genetic partial least squares have been used for small sets of structurally similar molecules. Machine learning techniques such as associative neural networks, kappa nearest neighbor random tree, random forest, and decision tree methods have been used to develop predictive models for large sets of molecules with varied structures. Planarity of the molecule (area/depth^2^ ratio) and molecular mass were found to be the critical requisites for molecules to be CYP1A2 substrates/inhibitors.

## 7. Protein-Based Studies Using Molecular Docking and Molecular Dynamics

The extensive ongoing research worldwide to better understand the oxidation of exogenous/endogenous substrates by CYP1A enzymes, and the efforts for the development of CYP1A inhibitors have led to the wide use of docking studies and molecular dynamic studies on the enzymes. To better understand the interactions of the substrate with the enzyme and the specificity of ligands for either CYP1A1 or CYP1A2, docking studies using computational molecular modeling tools has been the go-to method. The docked substrate-enzyme features that have been analyzed are the binding free energies, the interactions between the substrate and the residues lining the active site, and the distance between the heme iron and the substrate reacting group. The docking studies described on CYP1A enzymes employed different software including AutoDock, Surflex dock from Tripos SYBYL, the docking module from Molecular Operating Environment (MOE), etc. The binding scores reflect the binding free energy obtained using a functional form of various terms that describe different interactions including hydrogen bond interactions, hydrophobic interactions, ionic interactions, metal ligation, hydrophobic and polar/aromatic interactions, entropy, desolvations, etc. The value of the binding free energy obtained will depend upon the scoring method employed. The docking studies are validated if there is good correlation of the bioactivity to the docking score. Furthermore, the docking studies produce several binding poses. Some researchers employ 2 or more docking methods and find a consensus binding mode which is then used for scoring to obtain the binding free energy. Docking studies on CYP enzymes have used the optimal distance between the atom of oxidation on the ligand to the heme iron atom (4.0 Å to 7.5 Å) as the chief criteria for determining the best binding mode of the ligand.

Oxidation of aromatic hydrocarbons and polyaromatic hydrocarbons (PAHs) found in the environmental pollutants to carcinogens by cytochrome P450 enzymes is a major cause of concern. Docking and QSAR studies were performed by Gonzalez et al. on 37 representative compounds that consisted of PAHs, PAH diols, and heterocyclic aromatic compounds onto CYP1A1 [41]. They used a homology model built using SWISS-MODEL for CYP1A1 as the X-ray crystal structure of CYP1A1 was not reported at the time of this study. It was observed that hydrophobic interactions played an important role in ligand binding. The residues involved were Phe123, Phe224 and Phe258. It was also seen that the ligand orientations were favoring π-π interactions in an edge-to-face manner with Phe123, and offset stacked manner with Phe224 and Phe258. Heterocyclic aromatic carbon PAH diols exhibited binding orientations similar to those of PAHs with π-π interactions and hydrophobic interactions with the Phe residues and with the polar groups forming hydrogen bond interactions with polar amino acids in the active site such as Asn221, Ser122, or Asp320, respectively.

**Figure 6:**
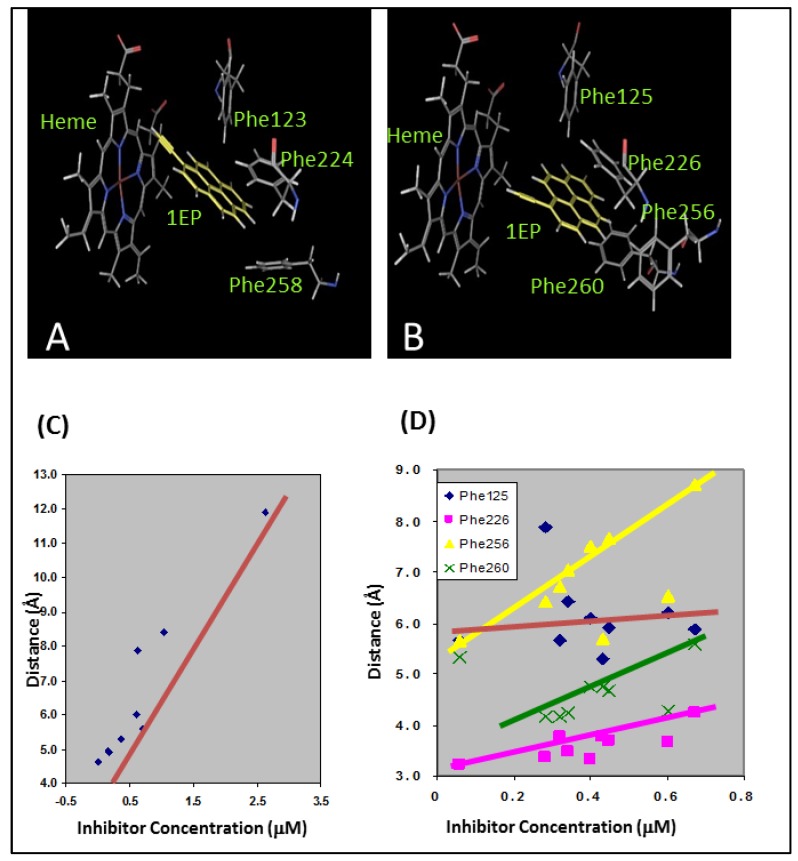
Docking studies showing the π-π interactions between the arylacetylenes and the enzymes (**A**) CYP1A1 and (**B**) CYP1A2. The docking studies also showed (**C**) the direct relationship between the inhibitory activity and the heme-acetylene moiety distance for CYP1A1 and (**D**) the relationship between the inhibitory activity and distance between centroids of aromatic rings of Phe residues and arylacetylenes for CYP1A2. [(**A**) and (**B**) reprinted from reference 52 with permission].

Arylacetylenes were developed by our group as time-dependent inhibitors of CYP1A1 and CYP1A2 [52]. These molecules are members of the PAHs with an acetylenic moiety as a substituent, which imparts the time-dependent inhibition via a reactive ketene intermediate. These molecules did not possess any polar groups and were incapable of hydrogen bonding interactions with the active site polar residues. All of the arylaceylenes made the critical edge-to-face and offset stacked π-π interactions with the Phe residues in the active site of the CYP1A enzyme (Figure 6A,B). For CYP1A1, the distance between the heme iron atom and the triple bond of the acetylene moiety determined the potency of the inhibitors (Figure 6C). In the case of CYP1A2, the π-π stacking interactions were found to be the most important determinants of the inhibition potency (Figure 6D).

**Figure 7:**
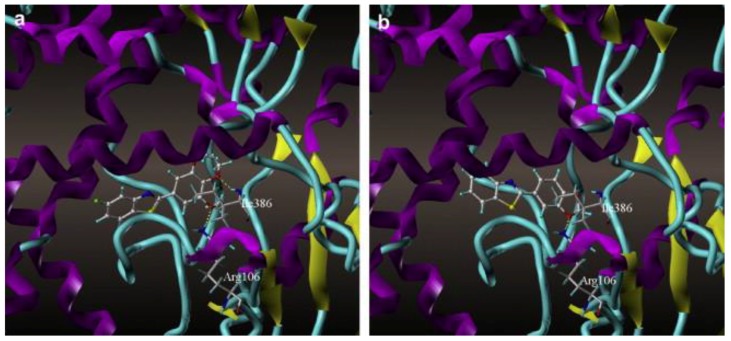
Adapted from reference [53] Model of 5-fluoro-2-(3,4,5-trimethoxyphenyl)benzo[d]thiazole (**a**) and 4-(benzo[d]thiazol-2-yl)benzene-1,2-diol (**b**) docked into the binding site of CYP1A1. Important residues are shown as sticks.

Benzoxazole and benzothiazole anticancer agents are known to inhibit CYP1A1. Surflex docking studies and CoMFA analysis of 48 derivatives of benzoxazoles and benzothiazoles were performed by Pan et al. [53] on the homology model of CYP1A1. Arg106 and Ile386 of CYP1A1 played important roles in forming hydrogen bonds with the oxygen atoms of these molecules. They concluded that the hydrogen bond interactions of these molecules with Ile386 are critical in determining the potency of inhibition for CYP1A1 (Figure 7). The binding free energy, CScores and the antitumor activity (pGI50) were compared for the docked structures of all 48 benzoxazole and benzothiazole derivatives. The pGI50 values ranged from 4.37 to 8.68, and the CScores varied from 4.47 to 7.54. The free energy of binding ranged from −6.07 kcal/mol to −10.38 kcal/mol. The authors found that the activity of the compounds corresponded well with the CScores.

Flavonoids are natural products that have antioxidative and antimutagenic properties thereby preventing several debilitating diseases including cancer, heart disease, and bone loss. Flavonoids are well known to inhibit several cytochrome P450 enzymes such as P450s 1A1, 1A2, 1B1, 2C9 and 3A4. Docking and QSAR studies were performed for several PAHs and flavonoids on the X-ray crystal structure of CYP1A2 and the homology model of CYP1A1 by Shimada et al. [54]. Several of docked flavones had hydroxyl groups at various positions of the aromatic rings. The ligand-interaction energies varied between −9.5 and 255 (U values, kcal/mol). A clear correlation between the U values and the IC_50_ values for inhibition of CYP1A1, CYP1A2 and CYP1B1 was not found. The binding orientations of these flavonoids differed between CYP1A1 and CYP1A2. In the case of strong inhibitors, the flavonoid B-rings docked close to the heme iron center of CYP1A2, and the π-π stacking of the favorably oriented flavonoid hydroxyl group with Phe226 was evidenced. Weak inhibitors favored an orientation that was different from that of the strong inhibitors for CYP1A2, in which the π-π stacking with Phe226 seen in strong inhibitors was absent for weak inhibitors. In the case of CYP1A1, all of the flavonoids docked in similar orientations in the active site. The studies indicated that the presence of a 5,7-dihydroxyl group in the A ring and a 3-hydroxyl group in the C ring of the flavone increased the inhibition potency for these enzymes. These studies also showed that the presence of 3′,4′-dihydroxyl group in the B-ring of the flavone could increase the inhibition potency.

Citrus fruits such as oranges, mandarins, and grapefruits are rich in flavonoids and coumarins that potently inhibit several CYP enzymes including CYP 1A1, CYP1A2, and CYP3A4. The components of grapefruit juice have been extensively studied for their interactions with several drugs and their role as chemoprotective agents. Furanocoumarin monomers, bergamottin and 6′,7′-dihydroxybergamottin (DHB) are potent inhibitors of CYP1A1 and CYP1A2 [55,56,57]. The flavonoid naringenin (NAR) isolated from grapefruit juice is a potent competitive inhibitor of CYP1A1. Espinosa-Aguirre et al. have studied the binding of DHB and NAR to human and rat forms of CYP1A1 through kinetic analysis and docking studies [58]. Kinetic analysis revealed species-related differences in the DHB and NAR inhibition of CYP1A1 by type and potency. DHB exhibited competitive inhibition of CYP1A1 in both species, but with an increased inhibition kinetics Ki for human CYP1A1 (55 μM) than for rat CYP1A1 (1.73 μM). NAR showed species variation in type of inhibition with competitive inhibition of human CYP1A1 (*Ki* = 489 μM) and mixed type inhibition for rat CYP1A1 (*Ki* = 0.17 μM and *K_I_* = 0.39 μM). Molecular dynamics simulations on the X-ray crystal structure of human CYP1A1 and homology model of rat CYP1A1 showed similar catalytic site fluctuations. Four critical residues in the binding site varied between the species- humanCYP1A1/ratCYP1A1: Ser116/Ala120, Ser122/Thr126, Asn221/Ser225, and Leu312/Phe316, and interactions with these differing residues by DHB and NAR could account for the differences in type and potency of inhibition. The docking scores for NAR and DHB for human CYP1A1 were −6.65 kcal/mol and −8.85 kcal/mol. The docking score for NAR with rat CYP1A1 was lower at −5.77 kcal/mol while the docking score for DHB with rat CYP1A1 was −8.05 kcal/mol, which is comparable to that of the human CYP1A1. The other interesting finding in these docking studies was the presence of two potential binding sites for DHB and NAR, the primary catalytic site and an adjacent secondary binding site below the primary site that was exposed to the solvent. This secondary binding site was much larger than the primary catalytic site in rat CYP1A1.

Our research group developed several flavone propargyl ethers that selectively inhibit CYP1A1 and CYP1A2 compared to CYP2A6 and CYP2B1 [45]. In that study, 3′-flavone propargyl ether (3′-PF) and 7-hydroxyflavone (7-HF) were also proved to be time-dependent inhibitors of CYP1A1. The rate constants of 3′-PF and 7-HF for maximal inactivation at saturation (*k*_inact_) were 0.09 and 0.115 min^−1^, respectively, and the concentrations required to produce one-half of the maximal rates of inactivation (*K_I_*) were 0.24 and 2.43 μM, respectively. Docking studies showed that these compounds exhibited multiple π-π interactions with the Phe residues in the active site of both CYP1A enzymes. For CYP1A2 enzyme, all of the compounds except for 5-hydroxyflavone were oriented in the active site in such a way that the hydroxyl group was closest to the heme of the enzyme. Even though the flavone propargyl ethers are longer molecules than the hydroxy flavones, their binding orientation in the enzyme active site showed the triple bond closest to the heme center with the signature π-π interactions with the active site Phe residues still maintained. It has been suggested that the unstable ketene intermediate formed by the oxidation of the triple bond could react with the neighboring Arg residue to form an amide bond, leading to the time-dependent inhibition of CYP1A1.

Coumarins are one of the classes of compounds that are known to be metabolized by several CYP enzymes including 1A1, 1A2, 3A4, 2A6 and some from the 2B subfamily. Based on the observation that the key metabolic site on the coumarin core structure is the 7-position, our group developed several substituted derivatives of 7-ethynylcoumarin that exhibit selective inhibition of CYP1A1 and CYP1A2 in a time-dependent manner [59]. Two different orientations were found for these 7-ethynylcoumarin derivatives in the CYP1A2 active site. 3-Phenyl substituted derivatives docked in such a way that the phenyl ring was facing the heme center leading to their behavior as competitive inhibitors. On the other hand, smaller derivatives with just alkyl substituents docked with the ethynyl group facing the heme that could explain the time-dependent inhibition of CYP1A2 by these molecules.

The advent of hormone replacement therapies in early/late menopausal women has been associated with a higher risk for breast cancer [60]. This has been attributed to the increased levels of hormones that are known procarcinogens [24,61]. Estrogen is metabolized by CYP enzymes (CYP1A1, CYP 1A2, CYP 1B1 and CYP 3A4) to its 2,3-dihydroxy and 3,4-dihydroxy forms. The 2,3-dihydroxy form is methylated to its non-carcinogenic form by cathecol-*o*-methyl transferase [62,63]. The 3,4-dihydroxy form is oxidized to its carcinogenic quinone form, which is a marker for human breast tumors, by peroxidases [24,61]. Yamamoto et al. have performed docking studies of 17β-estradiol on CYP1A1, CYP1A2 and CYP1B1 using Surflex Dock and the docked complexes were scored using an empirical scoring function based on the Hammerhead docking system [64]. They looked for binding modes that positioned the A-ring of the molecule within 5.5 Å of the heme iron atom. The binding mode of 17β-estradiol was identical for CYP1A1 and CYP1A2 and it differed from the one for CYP1B1. The accommodation of the 18-methyl group in the binding pocket was the deciding factor in determining the binding orientations of 17β-estradiol in the active sites of CYP1A1, CYP1A2 and CYP1B1 enzymes. These studies clearly indicate that CYP1B1 preferably oxidizes the 4-position while CYPs 1A1 and 1A2 prefer the 2-position. Docking studies of the keto form of estrogen, namely, estrone on the CYP1A1, CYP1A2, CYP3A4 and CYP1B1 were performed by Olah et al. using AutoDock Vina docking program [65]. Their results also indicated that the 2-position of estrone is primarily hydroxylated by CYP1A1 and CYP1A2, whereas, CYP1B1 preferably hydroxylated the 4-position.

During the development of a new class of quinone and anthraquinone inhibitors for CYP 1A enzymes, an ortho-methylaminoanthraquinone was found to be a time-dependent inhibitor of both CYP1A1 and CYP1A2 enzymes [36]. Docking studies on CYP1A1 and CYP1A2 showed that the methyl group was in close proximity to the heme, enabling the enzyme to oxidize the methyl group. Due to the presence of an amino group at the *ortho* position, the authors proposed that the hydrogen abstraction of the methyl group by an iron-oxo species would lead to the formation of a benzylic carbon radical intermediate. To better understand the nature of this proposed mechanism, the authors explored the inhibition characteristics of several polycyclic *ortho*-methylheteroarylamines [37]. Most of these compounds inhibited both CYP1A1 and CYP1A2 enzymes, with the linear planar molecules showing a marginal selectivity for CYP1A1. Four of these compounds showed time-dependent inhibition of CYP1A1 and not CYP1A2. Docking studies showed that the linear planar shape of molecules resulted in a better binding orientation in the active site of CYP1A1. Additionally, the molecules that had the aromatic methyl group in close proximity to the heme iron showed time-dependent inhibition of CYP1A1 with a mechanism similar to that of furafylline. Furafylline is a caffeine analog that selectively inhibits CYP1A2 (IC_50_ = 0.07 μM) over CYP1A1 (IC_50_ > 500 μM) and other CYP enzymes [66,67]. Furthermore , furafylline was found to be a mechanism-based inhibitor of CYP1A2 with a *Ki* of 23 μM and a *K_inact_* of 0.87 min^−1^. Docking studies have revealed that the furan ring interacts with Phe125 of CYP1A2 resulting in the 8-methyl group being directed towards the heme iron [68]. Isotope effects have clearly shown that the initiating step is the abstraction of a hydrogen from the C-8 methyl group of furafylline by P4501A2 [66]. It has been suggested that the loss of P450 observable spectroscopy could be the result of the formation of a reactive intermediate that may alkylate the heme prosthetic group or an amino acid in close proximity. Glycine scanning studies of the enzyme-ligand complexes for the compounds that were selective CYP1A1 time-dependent inhibitors showed that the ligands were making strong π-π interaction with the Phe123, Phe224 and Phe258 with a sandwich configuration contributing to the stabilization of the protein-ligand complexes [37]. π-π interaction of the ligand with Phe123 was the largest contributor for the complex stability. A similar glycine scanning study was performed for a set of carbazole analogs that are selective inhibitors of CYP1A2. Four residues in the active site contributed the most to the stability of the protein-ligand complex- Glu225, Phe226, Phe260 and Leu497. The ligand π-π interactions with the Phe residues were the strongest contributors for the stability of the complex. Additionally, Leu497 formed strong aromatic-aliphatic interactions by the positioning of this residue above a ligand aromatic ring. While Glu225 did not make any hydrogen bonds with the ligand, nonspecific aliphatic interactions contributed to the binding stability of the ligands.

**Figure 8:**
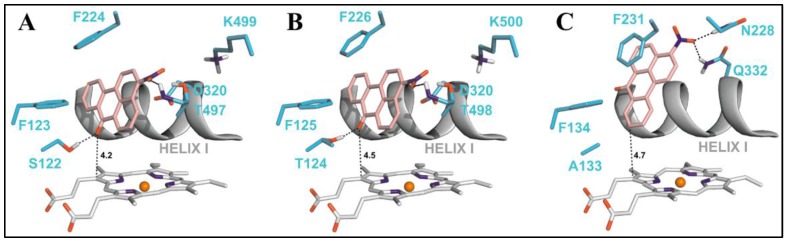
Adapted from reference [56]. The most favorable binding orientations of 3-NBA docked into the active site of CYP1A1 (**A**), 1A2 (**B**) and 1B1 (**C**). Hydrogen bonds between 3-NBA and the amino acid residues in active site residues are rendered as dashed black lines. 3-NBA (pink), heme (grey) and side chains of important amino acid residues (cyan) are rendered as bold sticks; iron ions as orange spheres.

Nitropolycyclic aromatic hydrocarbons (nitro-PAHs) are formed in the environment by the reaction of PAHs with nitrogen oxides in the atmosphere. The nitro-PAHs are metabolically activated by enzymes such as NADPH:quinone oxidoreductase (NQO1), NADPH:CYP oxidoreductase (POR), and CYP enzymes 1A1 and 1A2. Using theoretical and experimental approaches Stiborova et al. have shown that CYP1A1 and CYP1A2 efficiently activate the nitro-PAHs under anaerobic conditions to mutagens that bind to the DNA to form DNA adducts [69]. 3-Nitrobenzanthrone (3-NBA), found in diesel exhaust, is reductively activated to 3-aminobenzanthrone (3-ABA) by both NQO1 and CYP1A1/2 enzymes. The reductive activation of 3-NBA and the oxidative activation of 3-ABA, lead to the common intermediate N-hydroxy-3-nitrobenzanthrone (N-OH-3-ABA). To understand the reaction mechanisms of 3-NBA reduction by CYP1A1/2 enzymes, the authors performed docking studies using molecular modeling tools. A step-wise reduction mechanism (e^−^, H^+^, e^−^, H^+^) was proposed for the reduction of 3-NBA by CYP1A1/2. The binding orientation of 3-NBA to the CYP enzymes which allowed fast electron flow from the porphyrin ring of the heme cofactor was studied (Figure 8). This binding orientation was identical for both the CYP1A1 and CYP1A2 enzymes, wherein the nitro group of 3-NBA was in close proximity to the excellent proton donor threonine residues 497 and 498 in CYP1A1 and CYP1A2 which enables reduction of the compound. The same compound showed a different orientation in the active site of CYP1B1 in which the nitro group was oriented close to amidic side chains of Asn228 and Gln332 that are inferior proton donors. The estimated binding free energy for CYCP1A1 and CYP1A2 was −8.04 kcal/mol and −8.02 kcal/mol, respectively, which was much higher than the one for CYP1B1 (−7.73 kcal/mol).

Aristolic acid I (AAI) is a plant alkaloid procarcinogen metabolized by several cytochrome P450 enzymes, with CYP1A2 and CYP1A1 being the major enzymes involved in the O-demethylation of aristolic acid into the carcinogen 8-hydroxyaristolic acid I. Docking studies by Stiborova et al. [15] revealed that the binding free energy of AAI to CYP1A1 was −7.0 kcal/mol and to CYP1A2 was −7.7 kcal/mol. These binding free energies were far greater than the ones for CYP2C9 and CYP3A4 (−5.3 and −6.0 kcal/mol). This data clearly showed the preferred oxidation of AAI by CYP1A2 followed by CYP1A1 when compared to other P450 enzymes.

**Figure 9:**
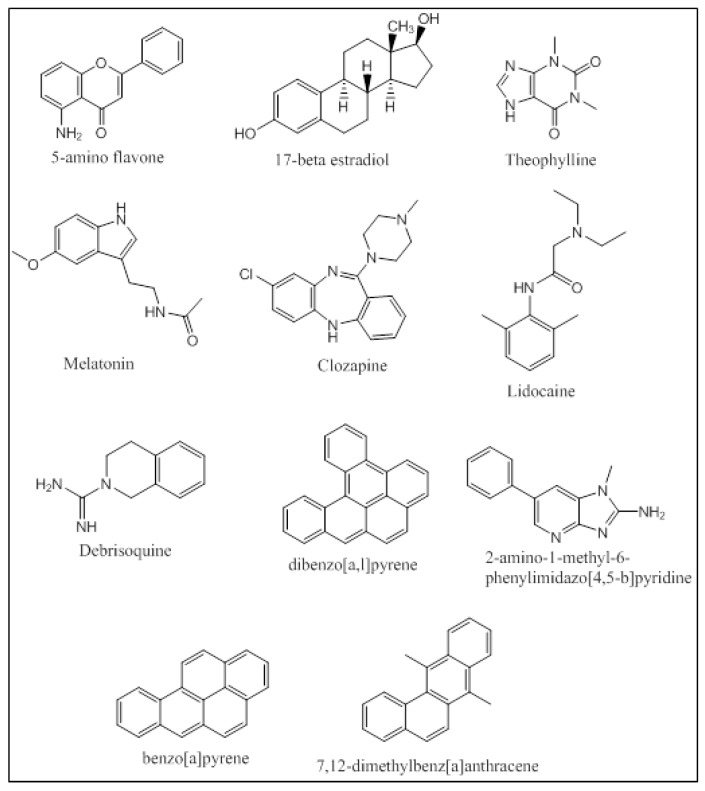
Structures of compounds studied by Kesharwani et al. [32] and Kahn et al. [57].

An integrated approach has been employed by Kesharwani et al. that applied molecular docking and molecular dynamic simulations of the protein-ligand complexes of a wide variety of P450 1A substrates [32]. Five classes of substrates were chosen based on their ability to be (i) preferably metabolized by CYP1A1 (5-aminoflavone and 5F-203); (ii) metabolized at different rates and percentages by CYP1A1/1A2/1B1 (17-betaestradiol and theophylline); (iii) metabolized at same rates and percentages by CYP1A1/1A2/1B1 (melatonin); (iv) preferably metabolized by CYP1A2 (clozapine and lidocaine); and (v) metabolized by all three enzymes CYP1A1/1A2/1B1 with specificity to CYP1A1 (debrisoquine) (Figure 9). Docking studies were performed using Glide, and the docked poses were ranked based on their binding orientation and distance from the heme to the site of metabolism on the ligand. Further MD simulation analysis of the docked complexes and calculation of the Molecular Mechanics Poisson-Boltzmann Surface Area (MM-PBSA) binding free energies provided atomic level information on the protein-ligand interactions. The docking studies were in accordance with the in-vitro results trend in terms of the enzyme that the ligand was most active against. The MD simulations and the MM-PBSA calculations matched up well for 5-aminoflavone, 17-betaestradiol, debrisoquine, and theophylline. Khan et al. performed docking studies using AutoDock Tools 4.0 followed by MD simulations of the protein-ligand complexes for several environmental polycyclic aromatic procarcinogens such as dibenzo[*a,l*]pyrene (DBP), 7,12-dimethyl-benz[*a*]anthracene (DMBA), 2-amino-1-methyl-6-phenylimidazo[4,5-b]pyridine (PhIP) and benzo[*a*]-pyrene (BP) on several CYP enzymes including CYP1A1 and CYP1A2 (Figure 9). CYP1A1 was found to be the predominant enzyme that metabolized BP, DMBA and PhIP with high binding energies of −11.50 kcal/mol, −11.24 kcal/mol and −9.43 kcal/mol, respectively. The in-silico findings for PhIP was different from the published experimental data, and this variation was attributed to the variation in the pKa values of certain ionizable residues in the binding groove of the CYP enzymes [70]. Qiu et al. studied the molecular interactions of 12 ginger components (including gingerols, shogaols, gingerdiones, and isogingerols, etc.) that are actively metabolized by the CYP enzymes 1A2, 2C9, 2C19, 2D6 and 3A4. Ginger is one of the most widely used herbal dietary ingredients and is known to have several important pharmacological activities including the maintenance of the levels of lipid, blood glucose, thromboxane B and prostaglandin E2; inhibition of cytokines and chemokines; and blockage of Ca^2+^ channel. Nearly 128 compounds have been isolated from ginger with gingerols, phenylpropanoids and sesquiterpenes being the most pungent of them. Shogoal and gingerol are partially responsible for the antiemetic activity. Docking studies have shown that CYP2D6 plays a major role in the metabolism of ginger components while CYP1A2, CYP2C9, CYP3A3 and CYP2C19 also metabolize ginger components, but to a lower extent. The Discovery Studio 3.1 CDOCKER was used for performing the docking studies. The CDOCKER interaction energies ranged from 23.0 to 63.1 kcal/mol. Three residues of CYP1A2 (Gly316, Phe226, and Phe260) interacted with the ginger compounds. 10-Gingerol made a hydrogen bonding interaction with Gly316 (CDOCKER interaction energy of 61.3568 kcal/mol) and 4 ginger compounds (6-shogaol, 8-shogaol, 6-gingerdione and methyl-6-isogingerol) made 1 to 2 π-π stacking interactions with Phe226 and/or Phe260 (CDOCKER interaction energies of 55.9354, 58.6712, 59.0853 and 51.232 kcal/mol, respectively). The CDOCKER interaction energies from the docking studies indicated that hydrogen bonding plays a greater role in the affinity of these compounds to the binding site of CYP1A2 than the π-π stacking [71].

## 8. Photo Affinity Ligands (PALs) as Binding Site Probes

**Figure 10:**
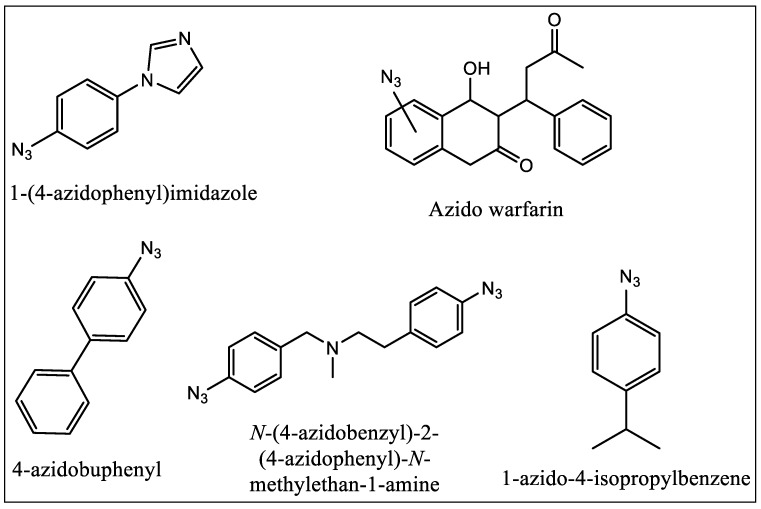
Structures of photo-affinity ligands used as binding site probes.

In 1962, Singh and Westheimer were the first who applied PALs to biochemical studies. PALs do not require any enzymatic activity to be utilized as active site probe. These ligands are in general very sensitive to the light and mostly are found to be stable in dark [72]. They can bind the chemically inert compound to the protein active site in the dark reversibly, and then irradiate the sample to produce the reactive moiety. In case of PALs with aliphatic side chains, the aliphatic group can be covalently labeled via photogeneration of a radical from the parent ligand. They do not always require nucleophilic amino acid residues to be in close proximity. The first example was reported by Swanson and Dus where they used tritiated 1-(4-azidophenyl)imidazole (Figure 10) [73] to successfully and quantitatively label P450cam. The compound was used as a general probe as N-phenylimidazole binds with high affinity to several P450 enzymes. The earliest success in photolabeling of P450 1A1 came in 1992 with the use of compounds having azo moiety on warfarin (Figure 10) [74]. The authors reported that they prepared different isomers of warfarin with azide group which was placed at different positions. In the same year Guengerich and his coworkers reported that they have successfully demonstrated the use of 4-azidobiphenyl (Figure 10) as a PAL of rat cytochrome P450 1A2 [75] and the covalent binding of the ligand following photolysis was shown in such a way that was PAL concentration-dependent and which increased with irradiation time.

In 1996, Strobel and coworkers first reported the use of bifunctional crosslinking PAL [76,77,78]. They used a benzphetamine analog having two azido groups which were placed at opposite ends. Strobel and coworkers used radiolabeled *p*-azidocumene (Figure 10) to identify the critical amino acid residue of P450 1A1 involved in binding of cumene hydroperoxide [76]. The experiments and studies performed by these and many other research groups have provided crucial data, which help in understanding the binding site of the CYP enzymes.

## 9. Conclusions

Computational molecular modeling studies have shed important insights into the binding of substrates to CYP1A1 and CYP1A2 enzymes. These studies have led to several important findings about the structural features imparting specificity and potency toward these enzymes. Ligand-based studies such as QSAR studies and structural meta-analysis studies have clearly shown that planar molecules are the best substrates for these enzymes. CYP1A1 shows a preference for linear planar molecules while CYP1A2 prefers triangular planar molecules. Protein-based studies have included docking studies and molecular dynamic simulations of protein-ligand docked complexes. The docking studies have shown the importance of edge-to-face and offset stacked π-π stacking of the ligand aromatic rings with the various Phe residues in the binding site of the CYP1A1 and CYP1A2 enzymes. These π-π stacking interactions were found to be the strongest contributors to the stable binding of ligands to CYP1A2. Docking studies have also shown that the optimum distance between the heme iron and the position of oxidation on the substrate should be between 4.0 Å and 7.0 Å. The positioning of polar substituents on the ligands in close proximity to polar residues in the binding site also favors good binding free energies for the complexes. The insights obtained by these studies will help researchers to design and develop highly specific inhibitors of CYP1A1 and CYP1A2 enzymes.
